# Size and Shape-Dependent Solubility of CuO Nanostructures

**DOI:** 10.3390/ma12203355

**Published:** 2019-10-15

**Authors:** Jindřich Leitner, David Sedmidubský, Ondřej Jankovský

**Affiliations:** 1 Department of Solid State Engineering, Faculty of Chemical Technology, University of Chemistry and Technology, Technická 5, 166 28 Praha 6, Czech Republic; Jindrich.Leitner@vscht.cz; 2 Department of Inorganic Chemistry, Faculty of Chemical Technology, University of Chemistry and Technology, Technická 5, 166 28 Prague 6, Czech Republic; sedmidub@vscht.cz

**Keywords:** CuO, nanoparticles, solubility, thermodynamics, Ostwald–Freundlich equation

## Abstract

In our theoretical study, the enhanced solubility of CuO nanoparticles in water saturated by air is predicted based on a simple thermodynamic model. CuO is considered in the form of nanoparticles with various shapes. The interfacial energy of a solid CuO/dilute aqueous solution interface was assessed by applying the average CuO surface energy and contact angle of a sessile drop of water. The equilibrium CuO solubility was calculated using Gibbs energy minimization technique. For the smallest spherical nanoparticles considered in this work (*r* = 2 nm), the solubility is significantly higher than the solubility of bulk material. In the case of cylindrical nanoparticles, the solubility increase is even more considerable. The CuO spherical nanoparticles solubility was also calculated using the Ostwald–Freundlich equation which is known to overestimate the solubility as discussed in this contribution.

## 1. Introduction

In recent years, an increasing interest in the research of transition metal oxides nanostructures emerged due to their unique properties and behavior differing from the bulk counterparts [1,2,3,4]. In this research area, it is very challenging to measure exactly particle sizes or particle-size distribution curves, as well as to experimentally determine the solubility of such nanoparticles [5,6]. Such results can then be compared with theoretical calculations based on thermodynamic modeling revealing the equilibrium background of these nanosizing effects.

CuO nanostructures (CuO-ns) are potentially toxic substances to many different organisms, such as plants, algae, bacteria, fish, and mammalian cells [7,8,9]. The mechanism of their toxicity is not definitely explained but strong evidence has been put forward that the CuO dissolution in an aqueous media plays a dominant role [10,11]. On the other hand, some observations indicate that nanoparticles themselves might be a source of toxicity due to enhanced formation of reactive oxygen species (ROS) [12,13,14]. It is well known that the solubility is size and shape-dependent [15,16], which has been observed for CuO [17,18,19] as well as for other oxide nanoparticles, e.g., ZnO [9,20] and CeO_2_ [21].

Enhanced solubility of nanoparticles is one of the numerous consequences of reducing the size of matter. Such behavior of small (nano-)particles has been predicted more than one century ago. Following Gibbs treatment of interfaces and their influence on equilibrium between solid particles and a liquid solution, the Ostwald–Freundlich equation could be derived in the form:

(1) $$\ln\frac{c_{i,r}}{c_{i,\infty }}=\frac{2\gamma_{\mathrm{sl}}V_{m,i}}{RTr}$$

where *V*_m_ is molar volume, *T* is absolute temperature, *R* is gas constant, *c_i,r_* and *c_i_*_,∞_ stand for equilibrium solubility of a substance *i* in the form of spherical particles of radius *r* and bulk material (*r* → ∞), respectively, and *γ*_sl_ represents the interfacial energy between solid particle and solution. Equation (1) holds for the ideal solution of *i* in a solvent and is based on the assumption that the interface energy *γ*_sl_ is not size-dependent. It was shown in our previous work [22], that this equation gives reliable results for spherical nanoparticles with a diameter greater than approx. 20 nm. For smaller nanoparticles, it predicts more enhanced solubility compared to the more advanced approach based on complex equilibrium calculation. This is due to the fact that the Ostwald–Freundlich equation—Equation (1), predicts the enhanced solubility as a consequence of the lower nanoparticles stability in comparison with bulk materials, and no response to changes in solution composition is considered. Moreover, the influence of the nanoparticles shape is also not considered in Equation (1). Based on a study published by Mistra et al. [19], CuO solubility is highly influenced by the shape of nanostructured CuO (spheres, rods, etc.). The size-dependence has also been observed in the case of bactericidal activity of CuO nanoparticles [23].

The aim of this work is to predict the size and shape dependence of CuO solubility in the form of spherical nanoparticles, nanorods, and nanoplates. The equilibrium solubility (composition of saturated solution in equilibrium with CuO) was calculated using Gibbs energy minimization technique considering thirteen Cu containing aqueous species and six solid Cu based compounds. The essential parameters for the calculations, namely interfacial energy between solid CuO and dilute aqueous solution, were estimated on the basis of the literature values of CuO surface energy and the contact angle of a water drop on the CuO surface.

## 2. Thermodynamic Description of CuO Dissolution

### 2.1. Dissolution of Bulk CuO

The solubility of bulk CuO in pure water has been investigated many times (for review see example [24]), and the equilibrium total Cu content (molality) in solution in the range 10^−7^–10^−6^ mol kg^−1^ was obtained (≈8 × 10^−6^–8 × 10^−5^ g CuO in one liter of solution) [25,26]. The solubility is significantly dependent on the pH of the solution achieving a minimum at around pH ≈ 10 and increases with both acidity and alkalinity increase of the solution. Cu^2+^(aq) is the dominant aqueous copper species in a neutral and acidic solution, while a variety of hydrated Cu(OH)*_n_*^(2−*n*)−^ (aq) species (products of Cu^2+^(aq) hydrolysis) are present in alkaline solutions. The solubility is also affected by the presence of some other complexing species, for example, Cl^−^, CO_3_^2−^, or PO_4_^3−^. Due to the carbonate ion or dissolved carbon dioxide, solid alkaline carbonates, namely malachite Cu_2_(OH)_2_CO_3_ or azurite Cu_3_(OH)_2_(CO_3_)_2_, can precipitate from aqueous solutions [26,27]. Moreover, some complex aqueous species can be formed which changes the total copper content as well as the copper speciation in the solution.

To calculate the equilibrium solubility of CuO in pure water and aqueous solutions, the non-stoichiometric method based on minimization of the total Gibbs energy of the system on the set of points satisfying the material balance conditions was used. The calculation algorithm and the computer program CHEMEQ were described elsewhere [28]. The liquid phase was considered as a dilute solution of 19 species in water (see Table 1). Chemical potentials of the solvent and the solutes were expressed with respect to Raoultian (pure substance) and Henrian (hypothetical ideal solution of the relevant solute in water at unit molality) standard states, respectively. To express the non-ideal behavior of the dilute aqueous solution, the Davies equation for solutes activity coefficients *γ_i_* was used [29]:

(2) $$\log\gamma_{i}=-0.509\;z_{i}^{2}\left(\frac{\sqrt{I_{m}}}{1+\sqrt{I_{m}}}-0.3I_{m}\right)$$

where *z_i_* is the ion charge and the Debye–Hückel constant 0.509 applied for *T* = 298.15 K and the ionic strength on the molal basis *I*_m_ is defined as:

(3) $$I_{m}=\frac{1}{2}\sum_{i=1}^{N}m_{i}\;z_{i}^{2}$$

where *m_i_* is molality.

The gas-phase containing N_2_, O_2,_ and CO_2_ were considered to be in equilibrium with the aqueous solution. The oxygen partial pressure *p*(O_2_)/*p*^o^ was fixed at 0.21 (standard pressure *p*^o^ = 100 kPa) and partial carbon dioxide partial pressure *p*(CO_2_)/*p*^o^ at 4 × 10^−4^. Apart from CuO, five other solid substances, namely Cu_2_O, Cu(OH)_2_, CuCO_3_, Cu_2_(OH)_2_CO_3,_ and Cu_3_(OH)_2_(CO_3_)_2_ were included in the calculations.

The values of standard Gibbs energies of formation (Δ_f_*G*^o^) were used as input thermodynamic data. The values for Cu aqueous species as well as for solid Cu compounds were adopted from the recent assessment by Puigdomenech and Taxén [30], while all other aqueous species, liquid water, and gaseous O_2_ and CO_2_ were adopted from the NBS tables [31].

### 2.2. Dissolution of CuO Nanostructures

Enhanced dissolution of nanostructured solids is due to the interface effect, which results in lower thermodynamic stability of materials with large specific interface area in comparison with bulk materials. As the first approximation, the enhanced solubility of spherical nanoparticles can be simply calculated using the Ostwald–Freundlich equation—Equation (1). As discussed later, this equation predicts a higher solubility for small nanoparticles (diameter under approx. 20 nm) than a more advanced approach based on complex equilibrium calculation taking into account the mass exchange between the solid and solution phase.

A simple procedure for equilibrium calculation in nanosystems was presented recently [32] and this approach was used for the demonstration of size/shape-dependent solubility of ZnO nanoparticles [22]. The interface effect can be simply treated by redefinition of the chemical potential of a solid substance in the form of nanoparticles surrounded by a liquid (fluid in general) phase. It is applicable for nanoparticles of general shape whose chemical potential *μ*^np^ can be expressed as:

(4) $$\mu^{\mathrm{np}}=\mu^{\mathrm{bulk}}+\gamma_{\mathrm{sl}}V_{m}{\left(\frac{dA}{dV}\right)}_{\mathrm{np}}=\mu^{\mathrm{bulk}}+\alpha^{\prime }\frac{2\gamma_{\mathrm{sl}}V_{m}}{r_{\mathrm{eqv}}}$$

where the so-called differential shape factor *α*′ is defined as [32]:

(5) $$\alpha^{\prime }={\left(\frac{dA}{dV}\right)}_{\mathrm{np}}\frac{r_{\mathrm{eqv}}}{2}$$

and *r*_eqv_ = (3*V*_np_/4π)^1/3^ is the radius of a spherical nanoparticle with the same volume as a non-spherical one. The interfacial energy *γ*_sl_ is dependent on the composition of the surrounding solution but in the case of dilute solution, a constant value referring to the solid/solvent interface can be used.

The above-described calculation of bulk CuO solubility in pure water was extended to CuO nanoparticles of various shapes (spherical as well as cylindrical with different aspect ratios). The chemical potential of CuO is given as:

(6) $$\mu_{\mathrm{CuO}}^{\mathrm{np}}=\mu_{\mathrm{CuO}}^{\mathrm{bulk}}+\alpha^{\prime }\frac{2\gamma_{\mathrm{sl}}V_{m,\mathrm{CuO}}}{r_{\mathrm{eqv}}}=\Delta_{f}G^{o}\left(\mathrm{CuO}\right)+\alpha^{\prime }\frac{2\gamma_{\mathrm{sl}}V_{m,\mathrm{CuO}}}{r_{\mathrm{eqv}}}$$

with *α*′ = 1 for spherical nanoparticles. In the case of cylindrical nanoparticles with a height *h* and radius *ρ* with a constant aspect ratio *x* = *h*/*ρ*, the differential shape factor *α*′ is given as [32]:

(7) $$\alpha^{\prime }={\left(\frac{2}{9}\right)}^{1/3}\left[x^{-2/3}+x^{1/3}\right]$$

and *r*_eqv_ = *ρ*(3*x*/4)^1/3^. The shape factor values *α*′ = 1.145 and 1.726 are obtained for *x* = 2 and 20, respectively. The value of *V*_m,CuO_ = 12.207 × 10^−6^ m^3^ mol^−1^ was used for solid CuO [33]. As for the interfacial energy, the average value *γ*_sl_ = 690 mJ m^−2^ was estimated as described in the next section.

Equilibrium calculations are more complicated in systems where two (or more) solid substances (phases) can coexist. In such a case, various geometrical models are available [34]—two (or more) single-component particles or one two (or more) component particles with core-shell or Janus geometry. Some other parameters such as interfacial energies on solid/solid interfaces are indispensable for such calculations.

## 3. Estimation of the Interfacial Energy CuO/Aqueous Solution

The interfacial energy *γ*_sl_ at the CuO/aqueous solution interface can be estimated using the Young equation and the values of surface energy *γ*_sg_ of solid CuO and contact angle *θ* of the water drop on the CuO surface (pure water can be used as an approximation of a highly diluted aqueous solution):

(8) $$\gamma_{\mathrm{sl}}=\gamma_{\mathrm{sg}}-\gamma_{\mathrm{lg}}\cos\theta$$

The surface energy of liquid water *γ*_lg_ = 72.0 mJ m^−2^ was used for calculation [35]. Surface energies for various crystallographic planes of CuO with monoclinic crystal lattice have been calculated using an *ab-initio* approach [36,37,38]. The individual values (see Table 2) were averaged according to the formula [39]:

(9) $$\gamma =\frac{n}{\sum_{\left(hkl\right)}\frac{1}{\gamma_{\left(hkl\right)}}}$$

yielding the resulting surface energy 1106.7 mJ m^−2^. This value refers to vacuum surroundings, but for real conditions, one must consider surface energy lowering due to the adsorption of some species from the surrounding atmosphere on the solid surface (primarily water vapor during contact angle measurements of the water drop). Analyzing literature data on calorimetrically determined surface energies for hydrated and anhydrous surfaces for a number of oxides [40], one can estimate that this reduction is approx. 38% and so *γ*_sg_ for a hydrated CuO surface is ~690 mJ m^−2^.

As for the contact angle of a water drop on a copper oxide surface, a lot of measurements have been performed exclusively on thin nanostructured films and coatings [41,42,43,44]. The obtained values cover quite a large range from 10° to 165° according to the actual composition of those films (presence of Cu, Cu_2_O, or Cu(OH)_2_) and their condition (as prepared, aged, further treated, and so on). Moreover, contact angles show film thickness-dependence in some cases. In such a situation, we decided to use a neutral value of contact angle 90° (cos*θ* = 0) and thus *γ*_sl_ ≈ *γ*_sg_ = 690 mJ m^−2^.

## 4. Results and Discussion

The solubility of bulk CuO was calculated at *T* = 298.15 K and *p* = 101.325 kPa. The aqueous solution was considered as saturated with O_2_ and CO_2_ by contact with gaseous-phase with fixed partial pressures *p*(O_2_)/*p*^o^ = 0.21 and *p*(CO_2_)/*p*^o^ = 4 × 10^−4^. The results are summarized in Table 3.

Under the above-mentioned conditions and at *p*(CO_2_)/*p*^o^ = 4 × 10^−4^ (average concentration of carbon dioxide in air), the calculated CuO solubility is *m*(Cu)_tot_ = 7.74 × 10^−6^ mol kg^−1^ (0.616 mg CuO) at pH = 6.41 and no other solid Cu substances is stable. Malachite can be formed by Equation (10) in a carbonized aqueous solution

(10) $$2\;\mathrm{CuO}(s)+H_{2}O(l)+{\mathrm{CO}}_{2}(\mathrm{aq})={\mathrm{Cu}}_{2}{\left(\mathrm{OH}\right)}_{2}{\mathrm{CO}}_{3}\left(s\right)$$

At 298.15 K, the equilibrium value of CO_2_(aq) molality is *m*(CO_2_(aq)) = 1.07 × 10^−4^ mol kg^−1^ which corresponds to CO_2_ partial pressure *p*(CO_2_)/*p*^o^ = 3.15 × 10^−3^. Hence, since the CO_2_ activity considered in our calculation is one order of magnitude lower, malachite is not formed at these conditions. The calculated CuO solubility in the limiting case *p*(CO_2_)/*p*^o^ → 0, namely *m*(Cu)_tot_ = 1.10 × 10^−7^ mol kg^−1^ (8.75 × 10^−3^ mg CuO) at pH = 7.37, is in good agreement with the data from the literature [24,25,26].

Enhanced solubility of CuO was calculated first for spherical nanoparticles of radius *r* according to the Ostwald−Freundlich equation—Equation (1) which, after substituting the numerical values, assumes the form:

(11) $$\frac{c_{\mathrm{CuO},r}}{c_{\mathrm{CuO},\infty }}=\exp\frac{6.7958}{r/\mathrm{nm}}$$

**Figure 1:**
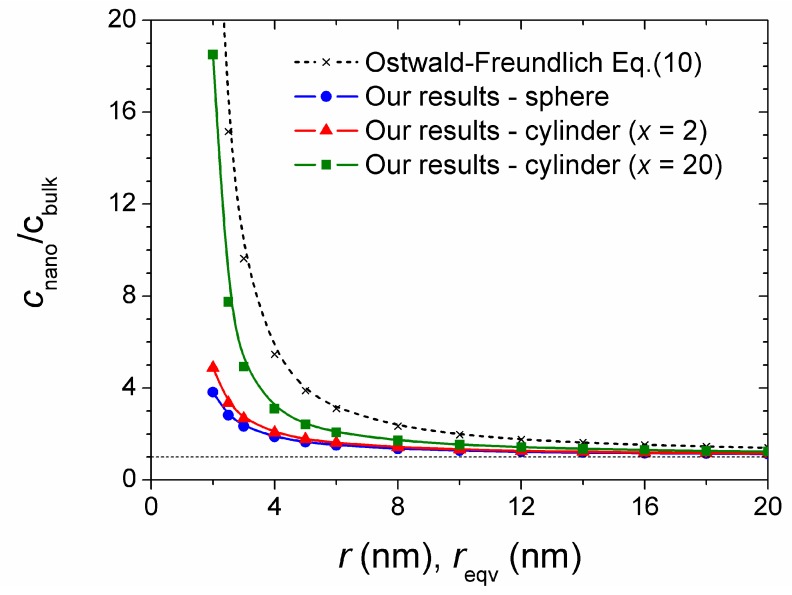
Calculated solubility of CuO−np in water at *T* = 298.15 K and *p* = 101.325 kPa.

The size-dependence of solubility is shown in Figure 1. For the smallest particles considered in the present study (*r* = 2 nm), such calculation gives almost 30-times higher solubility than for bulk CuO.

The results based on complex equilibrium calculations are summarized in Table 4 and plotted in Figure 1. It is obvious that there are serious discrepancies between the Ostwald−Freundlich prediction and our calculations. It is due to the fact that the calculation according to the Ostwald−Freundlich equation does not take into account the response of the solution to the reduced stability of dissolving nanoparticles. While for bulk CuO the resulting pH is 6.41 for the most soluble nanoparticles of radius *r* = 2 nm, pH = 6.91. Such a change in the final pH of the solution decreases the solubility of bulk CuO in approx. 6-times [24]. This effect is indeed not considered within the Ostwald−Freundilch equation.

**Figure 2:**
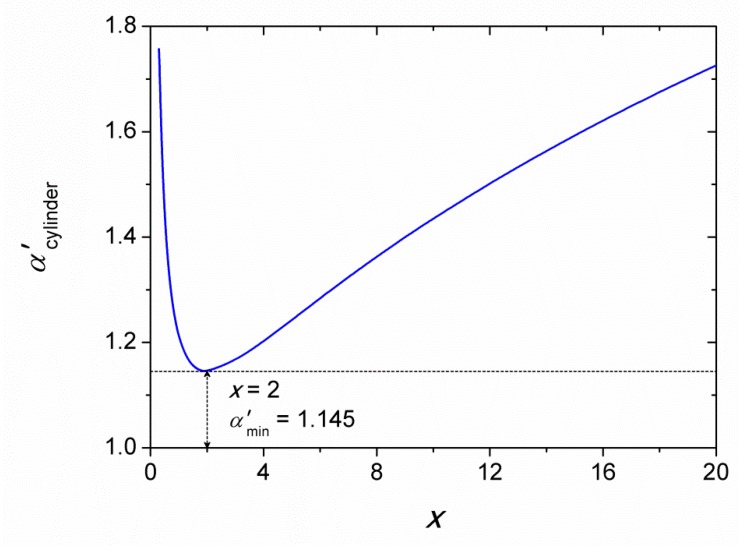
Dependence of differential shape factor for a cylinder on the aspect ratio *x* = *h*/*ρ* according to Equation (7).

As the shape-dependence of solubility is concerned, it is obvious that the larger the shape factor *α*′ the higher is the CuO solubility. It should also be noted, that the shape factor (and thus the solubility) increases for *x* < 2 as it follows from Figure 2.

Any quantitative comparison with experimentally determined solubility of CuO nanoparticles should be questionable due to various media in which the dissolution experiments have been carried out. The enhanced solubility of nanoparticles in comparison with bulk materials (microparticles) has been observed in a number of studies [17,18,19]. Wang et al. [11] have studied the dissolution of CuO nanoparticles (*r* = 20 nm) and have observed that approx. 0.25 mg L^−1^ of Cu released into the suspension of nanoparticles in 3% NaCl solution (320 mg L^−1^ CuO nanoparticles after 72 h exposition). Our calculated value 0.56 mg L^−1^ is almost twice greater but there is no evidence of the saturation of solution in the work of Wang et al. [11]. Misra et al. [19] have investigated dissolution of CuO nanospheres (diameter 7 nm) in 1mM NaNO_3_ solution (pH = 6.7). They have determined equilibrium solubility 1 mg Cu L^−1^ which is the same as our calculated value 1.71 = mg L^−1^ at pH = 6.69. They have also examined rod-shape CuO nanoparticles with length *h* = (40 ± 10) nm and diameter 2*ρ* = (7 ± 1) nm (*x* = 11.43, *α*′ = 1.484). The observed ratio of Cu content in the solutions was approx. 2.2 for nano-sphere and nano-rods. Our calculations for the same conditions give a lower value 1.3. It should be noted, that the same value of interfacial energy for CuO/solution interface was considered, but it is reasonable to suppose that the increase of surface-to-volume ratio for a cylinder might be partly compensated by enhanced exposition of lower energy surfaces in real nanoparticles. This would increase the stability of the nano-rods, reduce their solubility, and increase the calculated ratio of Cu content in solutions for nano-spheres and nano-rods.

## 5. Conclusions

In this work, we have used the theoretical approach to determine various CuO nanostructures (spheres or cylinders) solubility in an aqueous environment. The equilibrium CuO solubility was calculated using Gibbs energy minimization technique considering thirteen Cu containing aqueous species, out of which only Cu^2+^, CuOH^+^, Cu(OH)_2,_ and CuCO_3_ are significant in neutral or slightly acidic aqueous solutions. For the smallest spherical nanoparticles considered in this work (*r* = 2 nm), the solubility was 3.8-times the solubility of bulk CuO, suggesting significantly increased solubility due to nanosizing. The increase of solubility was even more considerable in the case of cylindrical nanoparticles. It is a well-known fact that copper oxide nanostructures are potentially toxic to many different organisms. The mechanism of their toxicity is not definitely explained but the strong evidence that the CuO dissolution plays the dominant role has been brought. Our study of CuO nanoparticles dissolution can be thus used for environmental issues, especially for water environment.

## Figures and Tables

**Table 1:** Substances considered in equilibrium calculation s and input thermodynamic data [30,31] (standard Gibbs energies of formation at *T* = 298.15 K and *p*^o^ = 100 kPa).

Substance	∆_f_*G*^o^ (kJ/mol)	Substance	∆_f_*G*^o^ (kJ/mol)
H_2_O(l)	−237.129	Cu_2_(OH)_2_^2+^(aq)	−285.1
H^+^(aq)	0	Cu_3_(OH)_4_^2+^(aq)	−633.0
OH^−^(aq)	−157.244	CuCO_3_(aq)	−501.5
O_2_(aq)	−16.4	Cu(CO_3_)_2_^2−^(aq)	−1048.98
CO_2_(aq)	−385.98	CuHCO_3_^+^(aq)	−532.08
CO_3_^2−^(aq)	−527.81	N_2_(g)	0
HCO_3_^−^(aq)	−586.77	O_2_(g)	0
Cu^+^(aq)	48.87	CO_2_(g)	−394.359
CuOH(aq)	−122.32	Cu_2_O(s)	−147.90
Cu(OH)_2_^−^(aq)	−333.05	CuO(s)	−128.29
Cu^2+^ (aq)	65.04	Cu(OH)_2_(s)	−359.92
CuOH^+^(aq)	−126.66	CuCO_3_(s)	−528.20
Cu(OH)_2_(aq)	−316.54	Cu_2_(OH)_2_CO_3_(s)	−902.35
Cu(OH)_3_^−^(aq)	−493.98	Cu_3_(OH)_2_(CO_3_)_2_(s)	−1431.43
Cu(OH)_4_^2−^(aq)	−657.48		

**Table 2:** Calculated values of surface energies *γ*_(*hkl*)_ for solid CuO [36,37,38].

(*hkl*)	*γ*_(*hkl*)_ (mJ m^−2^)		
	Ref. [36]	Ref. [37]	Ref. [38]
(111)	740	720	750
(−111)	860		890
(011)	930	910	940
(101)	1160		1170
(110)	1290	1180	1185
(010)	1370	1680	1485
(100)	2280	2240	1755
Average-Equation (9)	1094.6	1142.4	1083.2

**Table 3:** Calculated solubility of CuO in water at *T* = 298.15 K and *p* = 101.325 kPa (initial conditions: *n*^o^(CuO) = 1 mol, *n*^o^ (H_2_O) = 1 kg (55.5084 mol), *p*(O_2_)/*p*^o^ = 0.21).

*p*(CO_2_)/*p*^o^	*m*(Cu)_tot_ (mol kg^−1^)	pH	*I*_m_ (mol kg^−1^)	Dominant Aqueous Cu Apecies ^#^
4 × 10^−4^	7.74 × 10^−6^	6.41	2.29 × 10^−5^	Cu^2+^(95.8), Cu(OH)^+^(2.6), CuCO_3_ (1.0)
0	1.10 × 10^−7^	7.37	3.21 × 10^−7^	Cu^2+^(77.3), Cu(OH)^+^(19.9), Cu(OH)_2_ (2.5)

^#^ Number in parentheses means the percentage of the total Cu content in solution.

**Table 4:** Calculated solubility of CuO−np in water at *T* = 298.15 K and *p* = 101.325 kPa (initial conditions: *n*^o^(CuO) = 1 mol, *n*^o^ (H_2_O) = 1 kg (55.5084 mol), *p*(O_2_)/*p*^o^ = 0.21, *p*(CO_2_)/*p*^o^ = 4 × 10^−4^).

*r* or *r*_ekv_ (nm)	*m*(Cu)_tot_ (mol kg^−1^)		
	Sphere	Cylinder (*x* = 2)	Cylinder (*x* = 20)
2	2.959 × 10^−5^	3.779 × 10^−5^	1.433 × 10^−4^
3	1.811 × 10^−5^	2.072 × 10^−5^	3.818 × 10^−5^
4	1.450 × 10^−5^	1.595 × 10^−5^	2.395 × 10^−5^
5	1.275 × 10^−5^	1.373 × 10^−5^	1.871 × 10^−5^
6	1.171 × 10^−5^	1.245 × 10^−5^	1.601 × 10^−5^
8	1.054 × 10^−5^	1.104 × 10^−5^	1.327 × 10^−5^
10	9.911 × 10^−6^	1.028 × 10^−5^	1.189 × 10^−5^
12	9.509 × 10^−6^	9.798 × 10^−6^	1.106 × 10^−5^
14	9.232 × 10^−6^	9.473 × 10^−6^	1.050 × 10^−5^
16	9.031 × 10^−6^	9.236 × 10^−6^	1.011 × 10^−5^
18	8.877 × 10^−6^	9.056 × 10^−6^	9.893 × 10^−6^
20	8.755 × 10^−6^	8.914 × 10^−6^	9.579 × 10^−6^
bulk	7.741 × 10^−6^		
